# Navigating the Complex Landscape of Autism Spectrum Disorder: Challenges and Opportunities in Diagnosis, Treatment, and Supports

**DOI:** 10.2174/0113816128400132250716142203

**Published:** 2025-07-24

**Authors:** Arun Kumar Sharma, Sant Kumar Verma, Sidharth Mehan

**Affiliations:** 1 Division of Neuroscience, Department of Pharmacology, ISF College of Pharmacy, (Affiliated to IK Gujral Punjab Technical University, Jalandhar, Punjab, 144603, India), Moga, Punjab, India;; 2 Department of Pharmaceutical Chemistry and Analysis, ISF College of Pharmacy, (Affiliated to IK Gujral Punjab Technical University, Jalandhar, Punjab, 144603, India), Moga, Punjab, *India*

**Keywords:** Autism Spectrum Disorder (ASD), diagnosis, transition to adulthood, caregiver support treatment, challenges, DSM-5

## Abstract

Autism Spectrum Disorder (ASD) is a complex neurodevelopmental condition characterized by persistent deficits in social communication and interaction, as well as restricted, repetitive patterns of behaviour, interests, or activities. Despite advancements in our understanding of ASD, identification, screening, diagnosing, and treating this condition present significant challenges. This review article comprehensively examines the current diagnostic and treatment landscape for ASD, addressing key issues and opportunities for improvement. The diagnostic criteria for ASD, as outlined in the Diagnostic and Statistical Manual of Mental Disorders-5 (DSM-5), provide a framework for identifying the condition. Still, the heterogeneity of presentation and the presence of comorbidities contribute to diagnostic complexity. Early intervention is crucial for improving outcomes in individuals with ASD; however, accessing timely and appropriate interventions can be challenging. A diverse range of interventions exists for individuals with ASD, including behavioural therapies, pharmacological treatments, gene expression, and alternative therapies. However, the efficacy and accessibility of these treatments vary, and navigating the treatment landscape can be daunting for caregivers and clinicians alike. Moreover, due to the persistence of healthcare disparities, underserved populations face barriers to diagnosis and treatment. Transitioning to adulthood poses unique challenges for individuals with ASD, including finding employment and accessing support services. Additionally, ASD affects not only individuals diagnosed with the condition but also their families and caregivers. Addressing caregiver stress and burnout is essential for providing holistic care to individuals with ASD and their families. This review also identifies areas needing further research, such as personalized medicine and healthcare disparities, and discusses policy implications for enhancing ASD care and support. By highlighting research needs and policy considerations, this review aims to inform future efforts to improve ASD Screening, diagnosis, and treatment, ultimately striving to enhance outcomes for individuals with ASD and their families.

## 1 INTRODUCTION

ASD (Autism Spectrum Disorder) is a neurodevelopmental disorder that affects not only individuals with the disorder but also their families and society as a whole. With the increasing prevalence of ASD, it is crucial to clarify and demystify the factors in its diagnosis and intervention [1, 2]. The worldwide prevalence of ASD is estimated at approximately 1%, with the United States at about 1.7% and India at roughly 1.5% [3, 4]. Early diagnosis of ASD is essential to give the child appropriate therapies that can significantly improve a child's quality of life [5]. This review addresses the barriers to diagnosing and managing autism, as well as possible solutions [6]. While challenges remain, advancements in technology offer innovative solutions for diagnosis and support, community-based models create inclusion, and policy shifts are improving access to resources and services [7]. Better outcomes can be achieved with certain interventions; however, their effectiveness largely depends on accurate and timely diagnoses [8]. However, diagnosis of ASD remains a challenging and complex process despite the new knowledge that exists regarding research and clinical trials. Likewise, the therapeutic interventions should also be individualized according to the different symptoms of ASD and the unique needs of each person [9].

Early diagnosis is crucial, as intervention is most effective when initiated during key stages of development [10]. However, because of the variability of symptoms and the resemblance to other developmental disorders, diagnosis can be difficult [11]. Furthermore, access to specialized services and support remains limited, scarce, and uneven, thus contributing to inequity in diagnoses and care.

## 2 DIAGNOSTIC CRITERIA AND CHALLENGES

According to the developmental DSM-5, specific criteria must be met before diagnosing a person with ASD. To be diagnosed with ASD, an individual must demonstrate persistent deficits in social communication and social interactions across multiple contexts [12]. These deficits include Social-emotional reciprocity, Nonverbal communicative behaviours, and the ability to develop, maintain, and understand relationships [13]. Moreover, individuals must exhibit restricted, repetitive patterns of behaviours, interests, or activities like stereotyped movements, insistence that things remain the same, intense preoccupations, or unusual responses to sensory stimuli [14]. These symptoms must be present in the early developmental period, may not become fully manifest until social demands exceed capacities, and must cause clinically significant and marked impairment in social, occupational, or other important aspects of daily living [15]. These disturbances should not be better explained by intellectual disability or a global developmental delay [16].

The assessment of ASD is not always straightforward because children with the disorder exhibit a wide range of symptoms and often have co-occurring conditions [17]. ASD manifests with various severities, and symptoms can progress or regress over time, making diagnosis challenging [18]. Associated disorders include ADHD, anxiety, depression, and epilepsy, making assessment challenging for children with ASD [19]. Comorbidities often produce overlapping symptoms with other diseases’ symptoms, which causes incorrect or delayed diagnosis and treatment. Subcultural variations in symptoms are presented can also complicate diagnosis [20].

Furthermore, ASD is diagnosed through a behavioral approach rather than a single medical test; hence, diagnosis is a complex process that needs extensive observation and clinical expertise [2]. The ideal must possess ongoing social communication and interaction impairments in various situations. These domains include difficulties in social interaction, verbal and nonverbal communication fluency, and relationship comprehension and management [21]. Furthermore, learners must exhibit limited, perseverative behaviours, interests, or activities in areas like body movement, routine inflexibility, self-absorption, or peculiar responses to sensory stimuli [22]. Symptoms must be present in the early developmental period and cause significant impairment in social, occupational, or other important areas of functioning [23]. These disturbances should not be better explained by intellectual disability or global developmental delay.

### 2.1 Study Design and Methodology

This review article adheres to a systematic approach to identify, gather, and analyze the literature on Autism Spectrum Disorder (ASD). The study design was developed to ensure comprehensive coverage of the topic, transparency, and reproducibility. The methodology is outlined as follows:

### 2.2 Literature Search Strategy

A systematic search of peer-reviewed articles was conducted using major scientific databases, including PubMed, Scopus, Web of Science, and Google Scholar. Keywords and Boolean operators were employed to ensure a comprehensive search. The primary keywords included “Autism Spectrum Disorder (ASD),” “Diagnosis,” “Treatment,” “Caregiver Support,” “DSM-5,” “Early Intervention,” “Healthcare Disparities,” and “Transition to Adulthood.”Additional filters were applied to include studies published in English and within the last 20 years to ensure the inclusion of the most recent and relevant literature.

### 2.3 Inclusion and Exclusion Criteria

#### 2.3.1 Inclusion Criteria

Studies that focused on ASD diagnosis, treatment interventions, caregiver support, healthcare disparities, and transition to adulthood were included. Articles presenting primary research, systematic reviews, meta-analyses, and policy analyses were prioritized.

#### 2.3.2 Exclusion Criteria

Anecdotal studies, lacked sufficient methodological rigor, or focused on unrelated neurodevelopmental disorders were excluded.

### 2.4 Screening and Selection Process

The initial search results were screened by titles and abstracts to identify potentially relevant studies. Full-text articles were retrieved for further evaluation.

Two independent reviewers assessed the articles to minimize bias. Discrepancies in selection were resolved through discussion or consultation with a third reviewer.

### 2.5 Data Extraction and Synthesis

Key data from selected articles were extracted, including study objectives, methodologies, findings, and conclusions.

Thematic analysis was performed to categorize findings under major themes: diagnostic challenges, early intervention strategies, treatment approaches (behavioral, pharmacological, and alternative), healthcare disparities, and transition to adulthood.

**Fig. (1):**
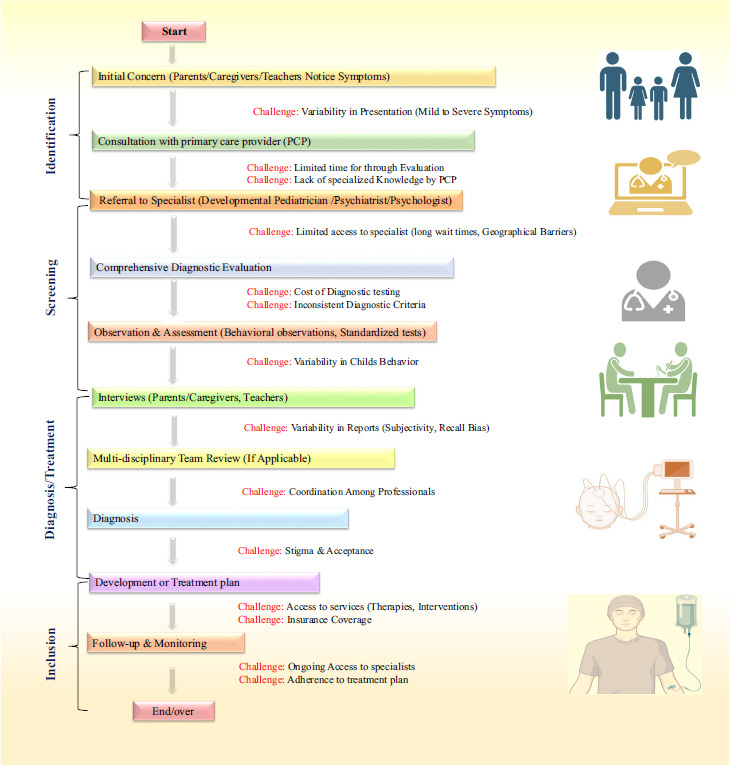
Flowchart depicting the screening, diagnostic, and treatment process for ASD, highlighting potential challenges at each step (*e.g*., limited access to specialists, variability in presentation).

**Fig. (2):**
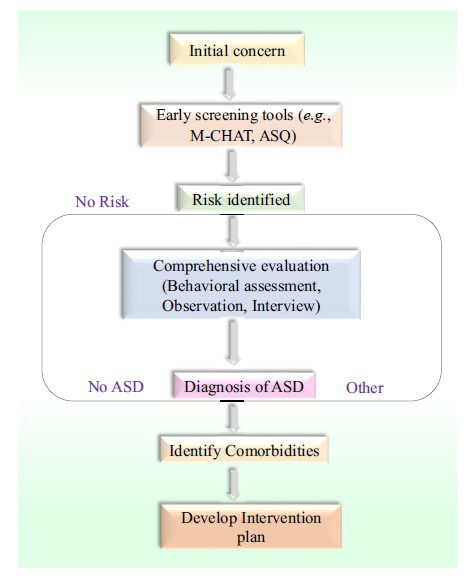
Diagrammatic flow chart: diagnostic process for ASD.

**Fig. (3):**
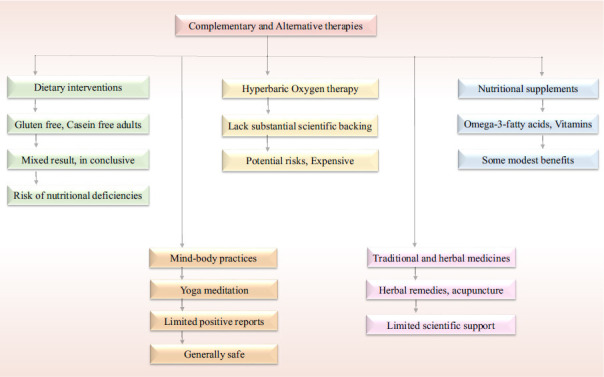
Complementary and alternative therapies for ASD.

**Fig. (4):**
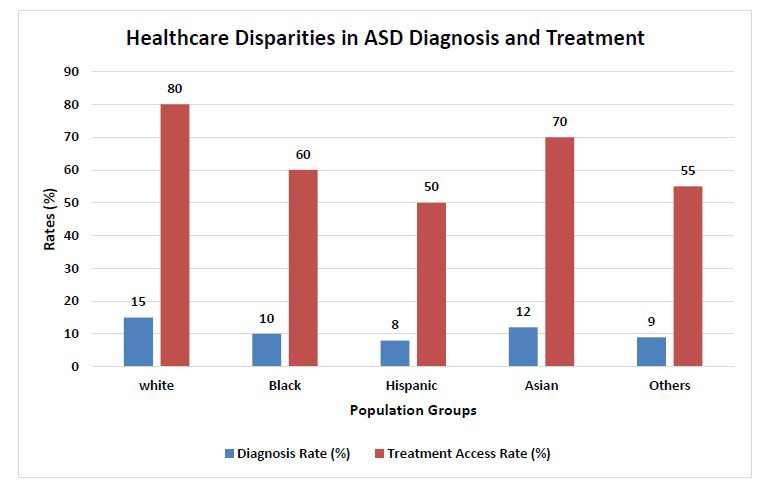
Graph illustrating healthcare disparities in ASD diagnosis and treatment across different populations.

Figures, tables, and flowcharts (*e.g*., Figs. **1**-**4** and Tables **1**-**8** in the manuscript) were created to visually represent the diagnostic and treatment processes, challenges, and disparities.

### 2.6 Quality Assessment

The quality of the included studies was assessed using established tools such as PRISMA (Preferred Reporting Items for Systematic Reviews and Meta-Analyses) guidelines.

Studies were evaluated for methodological rigor, sample sizes, statistical analyses, and reproducibility of results.

### 2.7 Ethical Considerations

As this study is a review of existing literature, no ethical approval was required. All data included in this review are publicly available through peer-reviewed publications.

Developmental screening measures are essential to facilitate the diagnosis of ASD at the earliest possible stage; thus, early intervention is facilitated (Figs. **1** and **2**). These tools include the Modified Checklist for Autism in Toddlers (M-CHAT), Ages and Stages Questionnaires (ASQ), and Autism Diagnostic Observation Schedule (ADOS) (Fig. **2**) [24]. They assist in determining which children are potential candidates for ASD and subsequent examination. However, these screening have limitations [25]. They may also be unable to identify the children with milder forms of ASD, and may not be as effective for very young children. They can sometimes yield false positive and false negative results, which may cause undue concern or a non-diagnosis [26]. Furthermore, for accurate interpretation of these tools, some professionals should be involved, and these tools may not be easily accessed in some regions. However, early screening still occupies an essential place in the diagnostics of ASD [27].

## 3 GENETIC AND ENVIRONMENTAL FACTORS

ASD does not have a single primary cause because of the complex nature of the disorder, the diversity in the manifestations of the disease, and the intensity of its symptoms [12]. It is hypothesized that both genes and the environment play a part in the development of the condition.

ASD has been strongly influenced by genetic factors, with studies showinghigh hereditary. For example, if one twin has been found to have ASD, there is a high likelihood that the other twin will indeed have the condition [28]. Different gene alterations and polymorphisms have been linked to ASD, particularly those of neurodevelopmental and synaptic-originated genes. Other genetic disorders that are linked with a high prevalence of autism include Fragile X syndrome as well as Rett syndrome (Table **1**) [29]. However, it has been established that the genetics of ASD are polygenic, meaning that multiple genes are involved, therefore making it almost impossible to identify a single gene that can cause the disorder. It has been found that multiple genes are implicated in ASD. ASD can sometimes be linked with a genetic disorder for some children, such as Rett syndrome or Fragile X syndrome [30]. Some of the other genes contain rare mutations that cause ASD along with other phenotypic features; these genes include ARID1 B, ASH1L, CHD2, CHD8, DYRK1A, POGZ, SHANK3, and SYNGAP1 (Table **1**) [31, 32].

Environmental factors also play a significant role in the development of ASD, interacting with genetic predispositions [33]. (Table **1**) [34]. Other factors having an association with the incidence of the disease include perinatal factors such as complications during birth, low birth weight, premature birth, and Other factors that serve as postnatal aspects that may affect the chances of developing ASD, including exposure to heavy metals and air pollutants as well as poor nutrition (Table **1**). However, no single environmental factor has been definitively proven to cause ASD. Researchers continue to investigate whether factors such as infections during pregnancy, certain medications, pregnancy complications, or exposure to air pollutants contribute to ASD risk [35, 36].

For instance, a child who has a genetic predisposition to ASD may be more susceptible to environmental influences, such as prenatal exposure to toxins or complications during delivery, which can trigger or exacerbate the condition [37]. These multiple interactions underscore the importance of considering both genetic and environmental factors when investigating the causes of ASD and developing effective prevention and treatment strategies [38].

## 4 EARLY INTERVENTION STRATEGIES

Early intervention in ASD is widely recognized as highly beneficial for both developmental progress and long-term outcomes (Fig. **1**) [51]. Research suggests that interventions are most effective when initiated during toddler and preschool years, as the developing brain is especially responsive to changes in cognition, communication, and social abilities at this stage. Studies have shown that early intervention can reduce the severity of autistic symptoms, improve academic performance, enhance quality of life for both the child and family, and promote greater independence and productivity [52].

EIPs for Early Intervention of Toddlers and Preschoolers with ASD. As of the moment, there are numerous resources outlining evidence-based practices tailored to young children with ASD. All these interventions aim to target the severity of social communication impairments, pragmatic functioning, and restrictive and repetitive behavioural patterns [53].

### 4.1 Applied Behavior Analysis (ABA)

ABA is one of the most extensively researched and widely used interventions for young children with ASD. It is a structured, evidence-based approach that uses principles of learning and motivation to increase positive behaviors (such as communication and social skills) and decrease challenging behaviors (such as tantrums or aggression) (Table **2**) [54]. ABA programs are highly individualized and often involve intensive, one-on-one therapy sessions. Caregiver involvement and training are key components to generalizing skills across settings [55].

### 4.2 Early Start Denver Model (ESDM)

ESDM is a comprehensive, play-based intervention for children aged 12-48 months with ASD. Rooted in ABA principles, ESDM emphasizes building positive relationships, fostering social engagement, and following the child’s lead during play [56, 57]. The approach integrates developmental and behavioral techniques within natural routines and includes active parental participation. ESDM has demonstrated effectiveness in improving communication, cognitive abilities, and adaptive functioning in young children with ASD [58].

### 4.3 Pivotal Response Treatment (PRT)

PRT is a naturalistic, play-based intervention derived from ABA. It targets pivotal areas of development, such as motivation, self-management, and social initiations, which can lead to widespread improvements in communication and social skills [59, 60]. PRT is highly child-centered, using the child’s interests and choices to drive learning and promote engagement. Family members are trained to implement PRT strategies in daily routines, supporting generalization of skills. Research indicates that children who begin PRT before age five often achieve significant gains in verbal communication and social interaction [61].

### 4.4 Speech and Language Therapy

Speech-language therapy is a fundamental component of early intervention for ASD, focusing on enhancing both verbal and nonverbal communication skills [62]. Therapists use individualized exercises and play-based activities to help children develop language, understand gestures, and improve their ability to express needs and interact socially. Positive reinforcement and the use of motivating objects or activities are common strategies to encourage communication (Table **2**) [63].

### 4.5 Occupational Therapy (OT)

OT addresses the development of fine and gross motor skills, sensory processing, self-care, play, and participation in daily routines [64]. OT interventions are tailored to each child’s strengths and challenges, often using play-based activities to build skills in areas such as dressing, feeding, handwriting, and social participation. Sensory integration techniques may be included to help children manage responses to sensory stimuli [65].

### 4.6 Cultural and Linguistic Barriers

Cultural and linguistic barriers can significantly impact the effectiveness of autism interventions by influencing both access and engagement [66]. Interventions that are not adapted to a family’s cultural values or language may lead to misunderstandings, reduced participation, or even discontinuation [67]. For example, research with Hispanic families in the U.S. shows that simply translating materials into Spanish is insufficient; interventions must also reflect core cultural values, such as familism and respect, and may benefit from delivery methods like parent-to-parent training [68]. In some cultures, autism-related behaviors may be interpreted differently, resulting in underdiagnosis or a lack of appropriate support [69]. Linguistic mismatches between providers and families can make it difficult to communicate goals and strategies, especially when families have limited proficiency in the interventionist’s language [70, 71]. For instance, if speech-language therapy is delivered only in English to a family that primarily speaks another language, parents may struggle to reinforce skills at home, reducing the intervention’s impact [72]. Studies indicate that culturally responsive interventions, those tailored to the family’s language, traditions, and values, produce significant improvements in social-communication outcomes for children and mental health for caregivers, highlighting the importance of both linguistic translation and cultural adaptation for effective intervention [73].

Early intervention strategies such as ABA, ESDM, PRT, speech therapy, and occupational therapy are evidence-based approaches that can lead to significant developmental gains for toddlers and preschoolers with ASD. The most effective programs are individualized, involve families, and are delivered intensively during the early years when the brain is most receptive to change.

Nevertheless, successful utilization of early intervention has already been evidenced, though many families face some obstacles when it comes to utilizing it [74]. Common barriers include:

### 4.7 Limited Availability

This is due to the limited availability of human resources and programs that result in long waiting lists and/or delays in getting the services [75]. Cost: It is important to note that as the early intervention services are sometimes costly, some families cannot afford to pay for it due to inadequate insurance coverage [52].

### 4.8 Lack of Awareness

Some of the families may not even know about what constitutes early intervention or about the existing services [76, 77].

### 4.9 Geographic Barriers

The scores/level of stimulation can further be influenced by other factors, such as inadequate or a lack of access to specialized intervention services by families in rural/underserved areas.

### 4.10 Cultural and Language Barriers

The inefficiency of treatment solutions can also be attributed to multicultural viewpoints and language barriers [78].

## 5 BEHAVIOURAL AND EDUCATIONAL INTERVENTIONS

Several intervention strategies can be applied to individuals with ASD, and all of them target some aspects of the condition [79]. ABA is one of the most frequently reported and widely recognized interventions, focusing on the teaching of certain skills and preferred behaviours while using positive and negative reinforcement (Fig. **2**) [80]. Other behavioral interventions include CBT (Table **2**) and social skills training, which is suitable for individuals with ASD. Learning interventions are also necessary, particularly for school-age children with ASD, because these interventions include behavioural therapies [81]. It includes IEPS for learners with learning disabilities, special teaching methods, and techniques that will allow learners with learning disabilities to mix with other learners (Table **2**) [82].

As we know, ABA is the most popular and impactful behavioural therapy for individuals with ASD. It includes using principles derived from learning and behaviour theories to promote positive behaviour change and eliminate negative behaviour [83]. ABA is a form of behaviorism where skills are first divided into small, achievable goals, and then rewards are given each timethe individual advances toward the goal (Table **2**) [84]. Other behavioural therapies are pivotal response treatment, which addresses pivotal skill domains, including motivation and self-regulation, and discrete trial training, which involves structured teaching of skills followed by positive reinforcement [85]. These therapies are individualized for each patient and often can enhance communication, social, and adaptive skills [86].

School-based Early Childhood Interventions for students with ASD aim to implement an educational environment. Such interventions are IEPs, which are individual educational plans prepared by educators, therapists, and parents to equip a child for learning [87]. For instance, structured teaching (TEACCH) involves aspects like orderliness, visual support, and regular schedules to assist children with ASD in coping with school schedules. Social Stories and Peer-Mediated Interventions are also employed to supplement social awareness and peer communication (Table **2**) [88]. All of these educational interventions are designed to enhance learning for children and help to address their academic, social, emotional, and behavioural needs [89].

Barriers to the Implementation of Behavioral and Educational Interventions for children with ASD include a shortage of skilled professionals, high costs, inadequate insurance coverage, and inconsistent application across home, school, and community settings [90]. Additionally, tailoring interventions specific needs of every child and ongoing monitoring can be resource-intensive [91]. Overcoming these challenges requires collaboration among parents, educators, therapists, and policymakers to ensure effective and sustainable interventions are accessible [92].

## 6 PHARMACOLOGICAL TREATMENTS FOR ASD

Pharmacological treatments for ASD are primarily aimed at managing associated symptoms such as irritability, aggression, hyperactivity, and anxiety, rather than curing the disorder or addressing its core social and communication deficits [102]. Currently, the only FDA-approved medications for ASD-related irritability in children are the atypical antipsychotics risperidone and aripiprazole, which have demonstrated efficacy in reducing tantrums, aggression, and self-injurious behaviors. However, they are often associated with side effects like weight gain, drowsiness, and metabolic changes (Table **2**) [103, 104]. Other medications, though not specifically approved for ASD, are sometimes prescribed off-label; these include stimulants like methylphenidate for hyperactivity and inattention, selective serotonin reuptake inhibitors (SSRIs) such as fluoxetine and fluvoxamine for anxiety, repetitive behaviors, and mood symptoms, as well as mood stabilizers and anticonvulsants like valproate for mood regulation and seizure management [105]. Glutamate modulators, such as memantine, and other agents are also under investigation for broader symptom control [106]. However, pharmacological management in ASD is complicated by individual variability in drug response, often due to genetic and pharmacogenomic differences, along with the risk of side effects and the limited efficacy of these medications in addressing the core features of ASD (Table **3**).

Access to specialist care and consistent medication management can also be challenging, particularly in rural or underserved areas. In response to these limitations, recent research has focused on developing drugs that target underlying neurobiological pathways in ASD [107]. Promising approaches include neurotransmitter modulation with agents like arbaclofen (a GABA-B agonist) and mGluR5 antagonists to restore inhibitory-excitatory balance in the brain [108], as well as the use of neuropeptides such as oxytocin and vasopressin analogues to enhance social bonding and interaction, though results are still preliminary (Fig. **1**).

Additional strategies involve synaptic and signaling pathway modulation, such as targeting the mTOR pathway, histone deacetylase inhibitors, and other epigenetic modulators, to improve synaptic plasticity and neural connectivity, as well as anti-inflammatory and antioxidant therapies like Trolox to reduce neuroinflammation and oxidative stress [109, 110]. The future of ASD pharmacotherapy lies in mechanism-based, individualized approaches that incorporate genetic and biomarker information to tailor treatments [111]. Ongoing clinical trials are evaluating the efficacy and safety of these novel agents, which may, for the first time, address the core features of ASD rather than just associated symptoms [112]. In summary, while current medications for ASD primarily address irritability, aggression, and co-occurring symptoms, emerging therapies that target neurobiological mechanisms hold promise for more comprehensive and personalized treatment in the future [105].

### 6.1 Challenges of Current Medications

#### 6.1.1 Side Effects

Increase in weight, insomnia, and discomfort in the stomach, which people sometimes avoid discussing [119].

#### 6.1.2 Limited Efficacy

Not always used for specifically treating an individual’s communication problems or repetitive movements [120].

#### 6.1.3 Individual Variability

Reaction and tolerability can be defined as being distinctly individual [121].

### 6.2 Potential Future Therapies

#### 6.2.1 Glutamate Modulators

Select the neurotransmitter system primarily involved in the pathophysiology of ASD [122].

#### 6.2.2 Oxytocin and Vasopressin Receptor Antagonists

Research for enhancing social interaction and controlling impulses and behaviours [123].

## 7 COMPLEMENTARY AND ALTERNATIVE THERAPIES

Complementary and alternative therapies (CAM) for ASD encompass a broad range of interventions used alongside or instead of conventional medical care [124]. These treatments include special diets (such as gluten- and casein-free diets), nutritional supplements (like omega-3 fatty acids and vitamins), mind-body interventions (such as yoga and meditation), herbal and natural remedies, acupuncture, massage therapy, and hyperbaric oxygen therapy (Table **4**, Fig. **3**) [125].

There is little consistent or reliable evidence supporting the effectiveness of complementary and alternative therapies for ASD [126]. Some interventions, such as omega-3 fatty acids, have shown modest benefits in small studies targeting symptoms like hyperactivity and repetitive behaviors, but results are inconsistent and not conclusive [127]. The effectiveness of other treatments, such as gluten- and casein-free diets, has not been conclusively established, with studies showing inconsistent findings (Table **4**, Fig. **3**) [128]. Small studies suggest that some individuals with ASD may benefit from yoga and meditation in terms of stress reduction and emotion regulation, but large-scale clinical data are lacking [129]. Hyperbaric oxygen therapy remains experimental, with anecdotal reports of benefit but insufficient scientific evidence to support its use [130].

Safety is an important consideration with CAM approaches. While some treatments, such as certain nutritional supplements, are generally safe when used appropriately, others can pose risks [131]. For instance, restrictive diets can lead to nutritional deficiencies if not properly managed, and herbal remedies may interact negatively with conventional medications [132]. The unregulated nature of many CAM therapies and the lack of standardized dosages also raise concerns about safety and efficacy (Table **4**) [132].

## 8 ADDRESSING HEALTHCARE DISPARITIES

Inequalities in the diagnosis and accessibility of treatment services influence the quality of the care received by people with ASD [142]. These differences can be attributed to factors such as socioeconomic status, race, ethnic background, and geographical region [143]. Low-income and minority children, children from rural areas, and children from ethnic backgrounds are diagnosed at a later stage and receive fewer opportunities to access specialized care than children with affluent, urban, or majority backgrounds [144]. This may result in a poorer prognosis and limited opportunities for learning and socialization of children with ASD [145].

Individuals with ASD in underrepresented communities encounter several hurdles in accessing appropriate care [66]. A Lack of funds limits the likelihood that families can pay for assessments and further care when insurance is insufficient [146]. Differences in language and cultural backgrounds can cause misunderstandings and result in poor communication between doctors and families when it comes to the management of the patient’s care [147]. Furthermore, there may be a few local healthcare centres having professionals with adequate training and experience in ASD, which makes necessary services unavailable in rural and other low-population zones. The above barriers play a part in exacerbating the general health disparities of people with ASD [148].

Several strategies can help minimize healthcare inequalities in ASD [149]. Greater outreach efforts and educating the public about ASD can also contribute to changing such a perception and getting people to seek early evaluation and treatment for their children [150]. Extending insurance coverage to encompass comprehensive ASD services and making these services affordable can help alleviate financial barriers [151]. Healthcare professionals should receive training in cultural competence, and translation services should be providedfor families that are not fluent in English [152]. Further, improving the accessibility of autism-specific specialists, expanding Autism care through telemedicine, and providing incentives to attract professionals in these areas will also boost the availability of care [153]. The elimination of these factors is intended to realize an improved healthcare system for people with ASD [154].

Conclusions on the Aspects Resulting in Unequal Access to Healthcare among ASD Patients:

1. **Socioeconomic Status:** Limited health care and lack of insurance hinder the chances of early and regular diagnosis as well as subsequent therapy [155].
2. **Cultural and Language Barriers:** Especially as the Tribes and families have noted, misunderstanding by the workers and utilization of peer-to-peer communication strategies lead to undertreatment [156].
3. **Geographic Location:** The lack of a sufficient number of specialized health workers, especially in rural and hard-to-reach areas, poses a significant problem in service delivery [157].

Measures Which May Help Reduce Healthcare Inequalities in Patients With ASD:

1. **Public Awareness and Education:** Stigma, early detection, and intervention [154].
2. **Insurance Coverage and Affordability:** The development of a sufficient and easily accessible ASD service system [158].
3. **Cultural Competence Training:** Emphasizing the aspects of increasing communication as well as the level of care for clients from different backgrounds [159].
4. **Increasing ASD Specialists:** The strategies of managing healthcare by using telemedicine and rewarding people for working in shortage areas [160].

By addressing these factors and implementing the proposed strategies, we can work towards reducing healthcare disparities in ASD and improving the quality of care for all individuals with autism (Table **5**, Fig. **4**).

The bar chart illustrates disparities in the diagnosis and treatment of ASD among different population groups. It shows the percentage of individuals diagnosed with ASD (blue bars) and those with access to treatment (red bars) for each group.

- In the White population, 15% are diagnosed with ASD, while 80% have access to treatment [166].
- The Black population has a diagnosis rate of 10% and a treatment access rate of 60% [167].
- Among the Hispanic population, 8% are diagnosed, with 50% receiving treatment [168].
- The Asian population shows a diagnosis rate of 12%, with 70% accessing treatment [169].
- For other unspecified groups, the diagnosis rate is 9%, and the treatment access rate is 55% [170].

The chart reveals a significant gap between the diagnosis and treatment rates, with treatment access consistently higher across all groups, indicating disparities in early diagnosis and access to services.

## 9 TRANSITION TO ADULTHOOD

Many difficulties are associated with the progression into adulthood in the case of patients diagnosed with ASD [171]. Some difficulties include transitioning from a stiff school system to a more flexible society. This transition may lead to challenges such as limited opportunities to enroll in college or acquire a job because of social communication disability and a lack of essential support [172]. Furthermore, many people with ASD can never learn how to go out on their own in society, how to manage their funds, personal hygiene, and other skills required in daily life, making them more dependent on their families or caregivers [173]. Limited social contact is also a major concern as establishing and maintaining interpersonal connections can be problematic, result in loneliness, and affect one’s psychological well-being [174]. However, most adult services are not well coordinated or specialized for individuals with ASD, and many of them do not get the necessary help to enable them to be productive citizens in adulthood [175].

To control these barriers, vocational and independent living supports are vital [176]. This category includes job training and development, career guidance and assistance, and other issues related to finding and sustaining paid work for people with ASD [177]. Those supported employment programs that include on-the-job modelling and follow-up support can significantly help. Assistance for the maintenance of independence includes coaching in areas like the preparation of meals, handling of money, and personal hygiene, among others [178]. Services that include community residential support or supervised living could be a stepping-stone toward full autonomy [179]. Moreover, social skills training can help with overall social interaction and compliance problems, which are common among people with ASD and can lead to social isolation. It can be concluded that the availability of such support and its customization according to the needs of each client are critical to its success [180].

It is, therefore, very important to understand the best way to fill the gaps in services for transitioning young individuals with ASD [181]. Another significant area of deficiency involves transition planning for young people from adolescence to adulthood [182]. It is also important to develop transition programs that offer services from high school through the adult years to maintain support. The second gap relates to the lack of adult services compared to those offered to children. There is a need to integrate more adult services, such as mental health, social skills groups, and community integration [183].

Furthermore, there is a requirement for increased knowledge and education within service delivery to cater to the needs of adults with ASD. Policies that support funding and resources for adult ASD services can also assist in closing these gaps [184]. It is the collective responsibility of families, teachers, practitioners, and policymakers to establish a support structure that will enable users of ASD to have an easier transition to adulthood [185] (Table **6**).

## 10 FAMILY IMPACT AND CAREGIVER SUPPORT

ASD not only affects individuals but also their families and caregivers. The emotional and financial strain of caring for a loved one with ASD can be significant [192]. This section will examine the impact of ASD on families, highlight the importance of caregiver support services, and discuss strategies for addressing caregiver stress and burnout [193].

Caregivers of children with ASD often face significant challenges, including high levels of stress, difficulty accessing resources, and the need for specialized skills to support their child’s development. Several evidence-based caregiver support programs have demonstrated success in improving both caregiver well-being and child outcomes [194, 195]. For example, Project ImPACT is a parent-mediated intervention that empowers caregivers to use evidence-based strategies for enhancing their child’s social communication and play skills [196, 197]. Studies have shown that Project ImPACT not only improves children’s communication abilities but also increases caregiver adherence to intervention strategies and reduces parenting stress, with both in-person and online formats proving effective across diverse settings. Other notable programs include JASPER (Joint Attention, Symbolic Play, Engagement, and Regulation), which trains parents to facilitate joint attention and symbolic play, and RUBI Parent Training, which provides structured guidance for managing challenging behaviors [198, 199]. The WHO Caregiver Skills Training (CST) Program offers accessible, group-based training for families globally, while Social ABCs focuses on coaching parents to promote early communication [200]. These programs typically include coaching, modeling, feedback, and peer support, helping caregivers feel more competent and less isolated [201] (Table **7**). Access to such support services and resources is crucial for addressing the unique challenges caregivers face and for fostering positive developmental outcomes in children with ASD [185].

## 11 FUTURE DIRECTIONS AND RESEARCH NEEDS

**Fig. (5):**
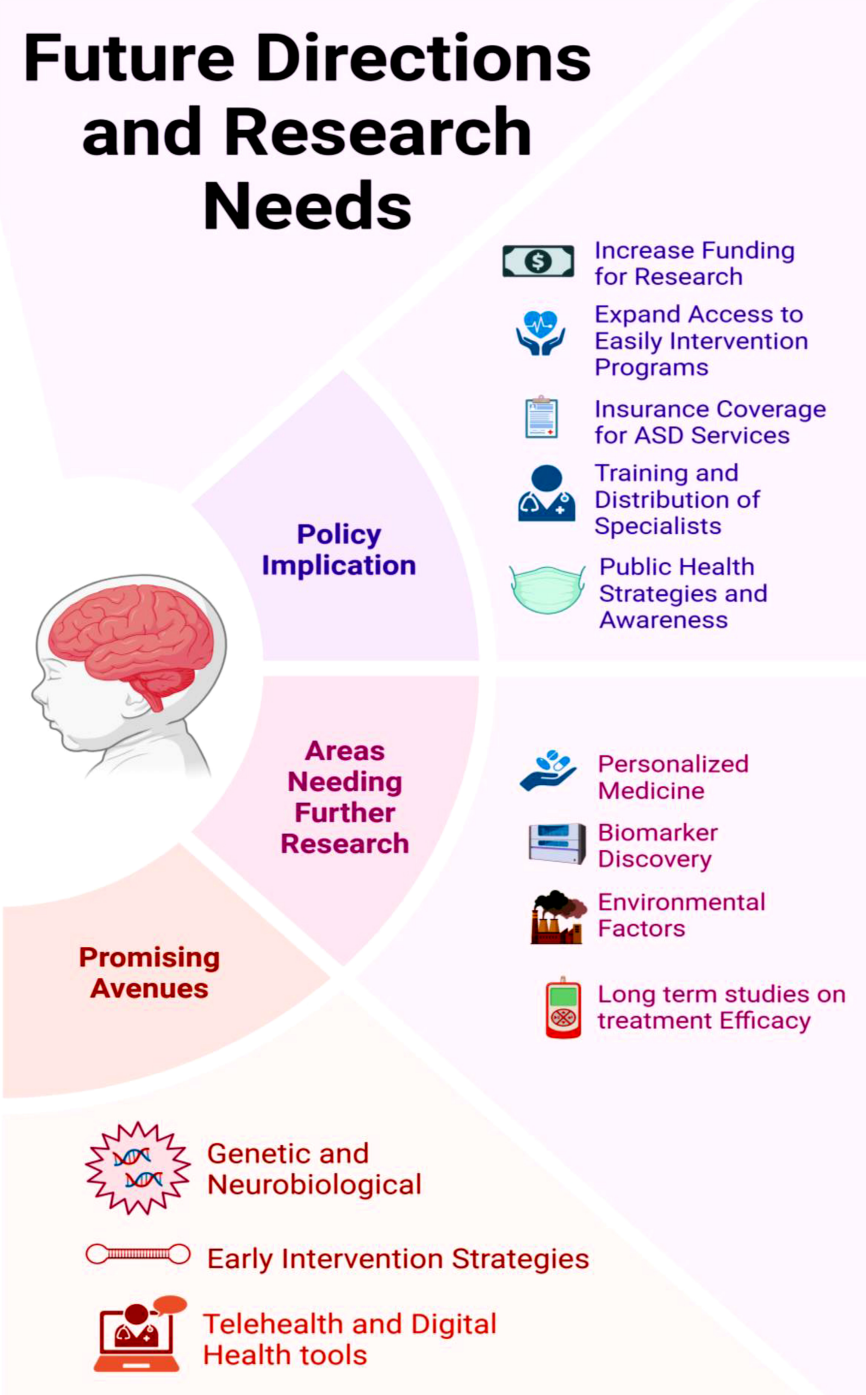
Future direction of research needs.

There are several research opportunities for enhancing ASD diagnosis and subsequent therapy avenues [215]. Genetic and neurobiological studies are helping to refine the knowledge base of ASD and its theoretical framework. This progress makes it easier to devise accurate diagnostics and selective therapeutic approaches at a later stage [216]. ACEs can be converted into favourable outcomes if early intervention based on the requirements of every client is applied [217]. Also, the use of technology will help increase the availability of diagnostic and therapeutic services by offering other modalities of diagnoses and treatments like telehealth and other digital health solutions where conventional services may not be available (Fig. **5**).

There is a need to advance research on various aspects of ASD to improve the diagnosis and treatment process [218]. The main concepts of this approach include understanding each patient as a unique individual with a unique set of genetic characteristics, biomarkers, and clinical indicators, and the potential for enhancing the effectiveness of treatments [219]. Biomarker discovery is another critical area, as identifying a new and stable biomarker is significant in enhancing diagnostic capabilities by making the same more accurate from the early stages [220]. There is a need for additional studies on the influence of environmental considerations on the occurrence and advancement of ASD. However, few multicenter, long-term randomized controlled trials have been conducted to assess new treatments' efficiency and side effects profile [221].

In this context, policymakers have a vital role in improving ASD care and support [222]. New policies should emphasize the funding of ASD research, especially in areas such as pharmacogenomics, individualized medicine, and biomarkers [131]. It is crucial to increase children’s access to early intervention services and to guarantee that these services remain cost-effective and reimbursable [223]. However, the geographic maldistribution of physicians can be decreased by policies aimed at the training and distribution of specialized human capital in healthcare [224]. Incorporating ASD intervention within population health and increasing advocacy and knowledge of the disorder can also lead to appropriate and proper support systems for those with ASD [225].

By pursuing these future directions and addressing the outlined research needs, we can improve the diagnosis and treatment of ASD, ultimately enhancing the quality of life for individuals with the disorder and their families (Table **8**).

### 11.1 Several Aspects of Autism that are Linked to Pregnancy

Arginine methylation, regulated by PRMTs and JMJD proteins, is a crucial post-translational modification essential for gene expression, DNA repair, and neuronal function [232]. While its role in cancer is well studied, arginine methylation is increasingly recognized as vital for CNS health, including in brain cancers, neurodegenerative, and neurodevelopmental disorders [233]. The Oxidation Resistance 1 (OXR1) gene, important for DNA repair, antioxidant defense, and neuronal protection, also regulates arginine methylation [234]. Loss-of-function mutations in OXR1 lead to cerebellar atrophy, epilepsy, developmental delay, and cognitive deficits [235]. Patient-derived cell and organoid studies show that OXR1 deficiency impairs cell survival, neural differentiation, and increases oxidative stress sensitivity by disrupting PRMT-mediated histone arginine methylation and its spatial-temporal regulation in the brain, underscoring the importance of arginine methylation and OXR1 in CNS development and disease [234].

Oxidative stress also negatively affects reproduction by disrupting DNA methylation, leading to epigenetic errors that can cause metabolic and psychiatric disorders [236]. Age and environmental endocrine disruptors further increase oxidative stress and methylation defects, with older men at higher risk of transmitting epigenetic abnormalities to offspring [237]. The one-carbon cycle links oxidative stress and methylation, supporting glutathione synthesis and homocysteine recycling, both vital for oocyte protection [238]. Assisted reproductive techniques may exacerbate methylation defects, while antioxidant supplements show inconsistent benefits. The bidirectional relationship between oxidative stress and methylation has significant implications for disorders like ASD, and recent advances in reproductive media composition, informed by this interplay, show promising clinical outcomes [239].

Homocysteine is a marker of disruptions in one-carbon metabolism, essential for embryonic and fetal development [240]. Elevated maternal homocysteine is linked to reduced first-trimester embryonic growth and lower birth weight, especially in IVF/ICSI with frozen embryo transfer and natural pregnancies, though not to fetal head growth [241]. The maternal periconceptional environment, *via* one-carbon metabolism, influences fetal neurodevelopment and risk of neural tube defects, cognitive issues, and ASD [242]. While maternal B6, B12, homocysteine, and choline generally do not affect fetal head growth, adequate first-trimester folate is associated with larger fetal head and cerebellar size, and deficiency with reduced brain volume [243]. Preconceptional folic acid and B vitamin-rich, low-homocysteine diets support fetal brain growth, though more research is needed to link maternal folate to neurodevelopmental disorders [244].

There is a growing need to emphasize prevention and actionable strategies. While folic acid supplementation is widely recommended for neurodevelopment, excessive intake, especially in individuals with MTHFR variants, may impair folate metabolism and methylation, increasing the risk of adverse outcomes such as ASD [245]. High levels of unmetabolized folic acid (UMFA), particularly in those with the MTHFR C677T polymorphism, can disrupt methylation and are associated with B12 deficiency and neurodevelopmental issues [246, 247]. Both insufficient and excessive FA during pregnancy may be harmful, with high maternal folate and/or B12 linked to higher ASD risk [248]. Given the limited effectiveness of current ASD treatments, prevention through personalized supplementation, considering genetic background, monitoring serum folate, and using alternatives like 5-MTHF, is essential [249]. The manuscript will be revised to highlight these prevention strategies and the need for further research on optimal folate intake during pregnancy, particularly for genetically susceptible populations, to ensure neurodevelopmental protection and safety [250].

## CONCLUSION

ASD presents significant challenges in both diagnosis and treatment, with late identification, often influenced by socioeconomic status and geographic location, limiting access to early, more effective interventions. Individual variability in medication response, frequent side effects, and the lack of treatments that address core ASD symptoms further complicate care. Access to specialists remains a major barrier, particularly in rural and underserved communities. Overcoming these obstacles requires an integrated approach: increased research funding, especially in pharmacogenomics and biomarker discovery; expanded, affordable early intervention services; culturally competent provider training; and incentives to attract specialists to areas of need. Integrating ASD care into broader health frameworks and raising public awareness can also foster a more inclusive environment. Early diagnosis is crucial for improving outcomes, but is often hindered by symptom variability, comorbidities, and shortages of trained personnel. Addressing these systemic gaps, along with supporting individuals during the transition to adulthood, is essential for equitable care. Ultimately, coordinated efforts among researchers, clinicians, policymakers, and communities are needed to advance best practices and improve the quality of life for individuals with ASD and their families. While ASD remains complex and challenging, ongoing research, innovation, and collaboration offer real opportunities for progress.

## Figures and Tables

**Table 1:** Genetic and environmental factors in ASD.

**Factor Type**	**Specific Factor(s)**	**Strength of Evidence**	**Summary of Evidence**	**References**
Genetic	Rare gene mutations (*e.g*., SHANK3, MECP2, UBE3A, FMR1)	Strong	Hundreds of risk genes identified; rare mutations can cause syndromic autism (*e.g*., Rett, Fragile X). Twin and family studies estimate heritability at 80-90%	[39, 40]
	Common genetic variants (SNPs, CNVs)	Strong	Common variants contribute significantly to ASD liability; GWAS studies support polygenic risk	[41]
	Epigenetic modifications	Moderate	Twin and epigenomic studies show epigenetic regulation impacts ASD risk, but mechanisms are still being elucidated	[42, 43]
Environmental	Advanced parental age	Moderate	Multiple studies show older parental age increases ASD risk, though the effect size is modest	[44]
	Prenatal exposure to air pollution (PM2.5)	Moderate to Strong	Meta-analyses link prenatal and early childhood PM2.5 exposure to increased ASD risk	[45]
	Maternal health conditions (*e.g*., diabetes)	Moderate	Some evidence links maternal diabetes and other health conditions to higher ASD risk, but causality is not fully established	[46, 47]
	Perinatal complications (*e.g*., hypoxia)	Moderate	Associations found, but confounding factors and causality remain under study	[48]
	Vaccines	No credible evidence	Large-scale studies have found no link between vaccines and ASD	[49]
	Chemical exposures (*e.g*., certain medications)	Weak to Moderate	Some associations have been reported, but the evidence is inconsistent, and causality is unclear	[50]

**Table 2:** Comparison table summarizing key features of different treatment approaches for ASD (*e.g*., ABA, medication, *etc*.), including their efficacy, limitations, and target areas.

**S. No.**	**Treatment Approach**	**Key Features**	**Efficacy**	**Limitations**	**Target Areas**	**References**
1.	Applied Behavior Analysis (ABA)	Structured, intensive, one-on-one therapy focusing on behaviour modification	Strong evidence for improving social, communication, and learning skills	Time-intensive, costly, and requires trained professionals	Social skills, communication, and adaptive behaviour	[93]
2.	Speech and Language Therapy	Targets communication skills and language development	Effective in improving communication abilities	Limited impact on non-communication-related symptoms	Communication, language development	[94]
3.	Occupational Therapy (OT)	Focuses on improving daily living skills and sensory integration	Helpful for enhancing motor skills and daily functioning	May not address core social and communication deficits	Fine motor skills, daily living activities, and sensory processing	[95]
4.	Social Skills Training	Teaches appropriate social interactions and skills	Beneficial for improving social interactions and relationships	It can be less effective if not tailored to individual needs	Social interactions, relationship building	[93]
5.	Cognitive Behavioral Therapy (CBT)	Focuses on identifying and changing negative thought patterns and behaviours	Useful for addressing anxiety, depression, and behavioural issues	Requires higher cognitive and verbal abilities	Anxiety, depression, behavioural issues	[96]
6.	Medication	Includes antidepressants, antipsychotics, and stimulants	It can help manage symptoms like irritability, aggression, and hyperactivity	Potential side effects do not address the core symptoms of ASD	Irritability, aggression, hyperactivity, co-occurring conditions	[97]
7.	Developmental, Individual-difference, Relationship-based (DIR/Floortime)	Emphasizes emotional and relational development through play	Promising for improving emotional and social capabilities	Less structured, requires active parental involvement	Emotional regulation, social skills, and parent-child interaction	[98]
8.	TEACCH (Treatment and Education of Autistic and Communication-Handicapped Children)	Structured teaching approach using visual supports	Effective for improving adaptive skills and independence	Requires significant training for implementation	Structured learning, adaptive behaviour, independence	[99]
9.	Dietary and Nutritional Interventions	Focus on modifying diet to improve symptoms	Anecdotal evidence for improvement in some cases	Limited scientific support, potential for nutritional imbalance	General well-being, dietary sensitivities	[100]
10.	Assistive Technology	Utilizes devices and software to aid communication and learning	Can significantly enhance communication and learning	Accessibility and cost require training to be used effectively	Communication, learning, and daily living skills	[101]

**Table 3:** Current pharmacological treatments.

**S. No.**	**Class of Drug**	**Medication**	**Targeted Symptoms**	**Route of Administration**	**Typical Dose**	**Side Effects**	**References**
1.	Stimulants	Methylphenidate	Hyperactivity, inattention	Oral	5-60 mg/day	Weight gain, sleep disturbances, gastrointestinal complications	[113]
2.	SSRIs	Fluoxetine, Fluvoxamine	Anxiety, depression, repetitive behaviors	Oral	10-80 mg/day	Nausea, sleep disturbances, weight changes	[114]
3.	Atypical Antipsychotics	Risperidone	Severe behavioral disorders, Irritability, and aggression	Oral	0.5-6 mg/day	Weight gain, drowsiness, increased appetite	[97]
4.	Atypical Antipsychotics	Aripiprazole	Severe behavioral disorders, Irritability, and aggression	Oral	2 - 15 mg/day	Weight gain, drowsiness, nausea	[115]
5.	Glutamate Modulators	Memantine, mGluR5 antagonists	Social behaviour, communication	Oral	5-20 mg/day	Dizziness, headache, constipation	[116]
6.	Oxytocin Analogues, Neuropeptides	Oxytocin, Vasopressin analogues	Social behaviour, emotional recognition	Nasal spray	24-48 IU/day	Nasal irritation, headache, drowsiness	[117]
7.	Vasopressin Receptor Antagonists	Balovaptan	Social behavior	Oral	10-40 mg/day	Nausea, headache, fatigue	[118]

**Table 4:** Overview of complementary and alternative therapies for ASD.

**S. No.**	**Therapy**	**Description**	**Evidence of Effectiveness**	**Safety Considerations**	**References**
1.	Dietary Interventions	Gluten-free, casein-free diets, ketogenic, Specific Carbohydrate Diet (SCD)	Mixed results, some surveys and case reports show improvements in behavior, GI symptoms, and cognition, especially with GFCF and SCD. Overall, evidence remains inconclusive, and individualized responses is common.	Risk of nutritional deficiencies (*e.g*., calcium, vitamin D, folate, B12); possible low bone density if not properly supplemented; regular monitoring advised.	[133, 134]
2.	Nutritional Supplements	Omega-3 fatty acids, vitamins (B12, D, C, folinic acid), minerals, melatonin	Some modest benefits, especially for hyperactivity, sleep, and certain behavioral symptoms; omega-3s may reduce hyperactivity and cluttering speech in young children. Folinic acid and B12 are rated highly by caregivers.	Generally safe if used appropriately, risk of overdose or interactions if unsupervised; monitor for adverse effects.	[135, 136]
3.	Mind-Body Practices	Yoga, meditation, mindfulness	Limited but positive reports on stress reduction, improving emotional regulation, and some behavioral improvements; evidence mainly from small studies and case reports.	Generally safe, low risk of adverse effects.	[137]
4.	Traditional and Herbal Medicine	Herbal remedies (*e.g*., ginkgo, curcumin), acupuncture	Limited scientific support, some anecdotal benefits reported, but robust clinical evidence is lacking. Potential for placebo effect.	Risk of drug interactions, allergic reactions, and side effects; the quality and purity of herbal products can vary.	[138, 139]
5.	Hyperbaric Oxygen Therapy	High-pressure oxygen chambers	Lacks substantial scientific backing; systematic reviews and controlled trials do not support efficacy for ASD core symptoms.	Potential risks include ear barotrauma, oxygen toxicity, and high cost; not routinely recommended.	[140, 141]

**Table 5:** Summary of factors and strategies for reducing healthcare disparities in ASD.

**S. No.**	**Factor**	**Description**	**Impact**	**Strategy**	**References**
1.	Socioeconomic Status	Financial constraints, lack of insurance	Limited access to diagnosis and treatment	Expand insurance coverage, provide subsidies	[161]
2.	Cultural and Language Barriers	Miscommunication, cultural misunderstandings	Suboptimal care	Cultural competence training, translation services	[162]
3.	Geographic Location	Shortage of specialized providers in rural/ underserved areas	Reduced access to necessary services	Increase ASD specialists, and use telemedicine	[163]
4.	Public Awareness and Education	Stigma, lack of knowledge about ASD	Delayed diagnosis, intervention	Public awareness campaigns	[164]
5.	Insurance Coverage and Affordability	High costs, inadequate coverage	Limited access to comprehensive care	Ensure affordable, comprehensive coverage	[165]

**Table 6:** Challenges and support needs for adults with ASD.

**S. No.**	**Challenges**	**Support Needs**	**References**
1.	Transition from the structured school environment to adulthood	Transition planning programs starting in high school	[186]
2.	Barriers to higher education and employment	Job training, career counselling, workplace accommodations	[187]
3.	Lack of independent living skills	Life skills training (cooking, budgeting, self-care)	[188]
4.	Social isolation and relationship difficulties	Social skills training, social groups, and mental health support	[189]
5.	Limited coordinated and specialized adult services	Expansion of adult services (mental health, community programs)	[190]
6.	Need for greater awareness and training among service providers	Training programs for service providers, policy advocacy for funding	[191]

**Table 7:** Outlining common challenges faced by families and caregivers of individuals with ASD, along with available support services and resources.

**S. No.**	**Challenges**	**Description**	**Support Services and Resources**	**Successful Caregiver Support Programs**	**References**
1.	Diagnosis and Early Intervention	Difficulty in obtaining a timely and accurate diagnosis, delays can impact intervention efficacy	Early intervention programs, developmental pediatricians, and local autism support groups	Project ImPACT (improves social communication, reduces parenting stress, builds peer support networks)	[202]
2.	Access to Healthcare Services	Limited availability of specialized healthcare providers and long wait times, especially in rural areas	Telehealth services, autism clinics, and community health centres	WHO Caregiver Skills Training (CST) (accessible online and in-person) teaches daily engagement skills, supports well-being)	[203]
3.	Educational Challenges	Finding appropriate educational settings and accommodations	Special education services, Individualized Education Programs (IEP), educational advocates	Social ABCs (parent-mediated, real-time coaching for communication and play in natural settings)	[204]
4.	Behavioural Issues	Managing challenging behaviours such as aggression, self-injury, or tantrums	ABA therapy, behavioural therapists, parenting workshops	RUBI Parent Training (teaches parents behavioral strategies using the ABC model, reduces challenging behaviors)	[205]
5.	Social Skills Development	Helping individuals with ASD develop social skills and make friends	Social skills training groups, peer mentoring programs, and recreational therapy	JASPER (hands-on parent training to improve joint engagement, play, and generalization to classroom settings)	[206]
6.	Communication Difficulties	Addressing verbal and non-verbal communication challenges	Speech and language therapy, augmentative and alternative communication (AAC) devices, communication apps	Social ABCs (focuses on communication skills and emotional regulation for both child and caregiver)	[207]
7.	Financial Strain	High cost of therapies, treatments, and special education	Government assistance programs (SSI, Medicaid), nonprofit organizations, grants, and scholarships	Caregiver support programs often include resource navigation and financial counseling	[208]
8.	Mental Health Concerns	Higher rates of anxiety, depression, and other mental health issues among individuals with ASD	Counselling and mental health services, support groups for caregivers, and respite care services	Project ImPACT and WHO CST (include stress management and psychological support components for caregivers)	[209]
9.	Caregiver Burnout	Physical and emotional exhaustion from caregiving responsibilities	Respite care, caregiver support groups, and mental health counselling	All listed programs include components to reduce caregiver stress and promote self-care	[210]
10.	Social Isolation	Feelings of isolation due to the demands of caregiving and a lack of understanding from others	Online forums, local autism support groups, family and caregiver workshops	Project ImPACT (peer network building), WHO CST (community-based, peer learning)	[211]
11.	Transition to Adulthood	Preparing for life after high school, including employment, independent living, and higher education	Vocational training programs, independent living skills programs, and transition planning services	Many caregiver programs provide transition planning modules or referrals	[212]
12.	Legal and Advocacy Issues	Navigating legal rights and obtaining appropriate services and supports	Special education advocates, disability rights organizations, and legal aid services	Caregiver support programs often include advocacy training and information sessions	[213]
13.	Sensory Sensitivities	Managing sensitivities to light, sound, touch, and other sensory inputs	Occupational therapy, sensory integration therapy, and environmental modifications	Social ABCs and WHO CST (teach strategies for managing sensory issues in daily routines)	[214]

**Table 8:** Summary of future directions and research needs in ASD.

**S. No.**	**Focus Area**	**Description**	**Importance**	**Example Strategies**	**References**
1.	Promising Avenues	Advances in genetic/neurobiological research, early intervention, and telehealth	Improves diagnosis, personalized treatment	Enhance diagnostic tools, tailor interventions, and expand telehealth	[226]
2.	Personalized Medicine	Tailoring treatments based on genetic/clinical profiles	Improves therapeutic outcomes	Develop personalized treatment plans	[227]
3.	Biomarker Discovery	Identifying reliable biomarkers for diagnosis	Enables earlier, accurate diagnoses	Invest in biomarker research	[228]
4.	Environmental Factors	Studying the role of environmental influences	Enhances understanding of ASD development	Conduct longitudinal environmental studies	[229]
5.	Long-term Studies	Researching the efficacy/safety of treatments	Ensures sustained benefits without adverse effects	Fund long-term clinical trials	[230]
6.	Policy Implications	Enhancing ASD care/support through policy changes	Increases research funding, access to care	Increase funding, expand early intervention programs, and improve insurance coverage	[231]

## LIST OF ABBREVIATIONS

AAC: Augmentative and Alternative Communication
ABA: Applied Behavior Analysis
ADOS: Autism Diagnostic Observation Schedule
ARID1B: AT-Rich Interaction Domain 1B (a gene associated with autism spectrum disorder)
ASD: Autism Spectrum Disorder
ASH1L: ASH1 Like Histone Lysine Methyltransferase (a gene linked to neurodevelopmental disorders, including autism)
ASQ: Autism Spectrum Quotient
CAM: Complementary and Alternative Medicine
CBT: Cognitive Behavioral Therapy
CHD2: Chromodomain Helicase DNA Binding Protein 2 (a gene associated with epilepsy and autism)
CHD8: Chromodomain Helicase DNA Binding Protein 8 (a gene linked to autism)
DIR: Developmental, Individual Differences, Relationship-Based Model
DSM-5: Diagnostic and Statistical Manual of Mental Disorders, Fifth Edition
DYRK1A: Dual-Specificity Tyrosine Phosphorylation-Regulated Kinase 1A (a gene associated with intellectual disability and autism)
EIPS: Early Intensive Behavioral Intervention
ESDM: Early Start Denver Model
FDA: Food and Drug Administration
Fragile X: Fragile X Syndrome (a genetic condition associated with autism)
GABA: Gamma-Aminobutyric Acid (a neurotransmitter involved in many neurological processes)
IEP: Individualized Education Program
M-CHAT: Modified Checklist for Autism in Toddlers
OCD: Obsessive-Compulsive Disorder
OT: Occupational Therapy
PCP: Primary Care Physician
PRT: Pivotal Response Treatment
SEs: Special Education Services
SHANK3: SH3 and Multiple Ankyrin Repeat Domains 3 (a gene associated with autism)
SSI: Supplemental Security Income
SSRIs: Selective Serotonin Reuptake Inhibitors
SYNGAP1: Synaptic Ras GTPase Activating Protein 1 (a gene associated with intellectual disability and autism)
TEACCH: Treatment and Education of Autistic and Communication Handicapped Children
